# Meaning-making behavior in a small-brained hominin, *Homo naledi*, from the late Pleistocene: contexts and evolutionary implications

**DOI:** 10.7554/eLife.89125

**Published:** 2025-09-04

**Authors:** Agustín Fuentes, Marc Kissel, Penny Spikins, Keneiloe Molopyane, John Hawks, Lee R Berger

**Affiliations:** 1 Department of Anthropology, Princeton University Princeton United States; 2 Department of Anthropology, Appalachian State University Boone United States; 3 Department of Archaeology, University of York York United Kingdom; 4 Centre for the Exploration of the Deep Human Journey, School of Anatomical Sciences, University of the Witwatersrand Johannesburg South Africa; 5 Department of Anthropology, University of Wisconsin Madison United States; 6 The National Geographic Society Washington DC United States; 7 The Carnegie Institution for Science Washington DC United States; Pennsylvania State University State College United States; Pennsylvania State University State College United States

**Keywords:** *Homo naledi*, human evolution, encephalization/cognition, mortuary behavior, meaning-making, Pleistocene, Other

## Abstract

Explorations in the Dinaledi Subsystem of the Rising Star cave system have yielded some of the earliest evidence of a mortuary practice in hominins. Because the evidence is attributable to the small-brained *Homo naledi*, these analyses call into question several assumptions about behavioral and cognitive evolution in Pleistocene hominins. The evidence from the Dinaledi Subsystem, and at other locations across the Rising Star cave system may widen the phylogenetic breadth of mortuary, and possibly funerary, behaviors. These discoveries may also associate the creation of meaning-making and increased behavioral complexity with a small-brained hominin species, challenging certain assertions about the role of encephalization and cognition in hominin and human evolution. We suggest that the hominin socio-cognitive niche is more diverse than previously thought. If true, technological, meaning-making activities, and cognitive advances in human evolution are not associated solely with the evolution of larger-brained members of the genus *Homo*.Evidence for complex behaviors associated with a small-brained hominin suggests that large brains are not solely responsible for the manifestation of human-like behavioral complexity.

## Introduction

Contemporary humans engage in shared meaning via vocal, visual, tactile, and scent communication, often involving patterned use of bodies, objects, and materials. Of the very broad range of behaviors that create embedded shared meaning, the only ones that persist in archaeological contexts are those that leave material traces. Examples include aspects of mortuary behavior, engravings or paintings, personal ornaments, and use of ochre or other pigments. Prior to this century, most researchers readily accepted that such material traces could be interpreted as products of meaning-making behaviors when associated with *Homo sapiens*. But when such traces were associated with Neanderthals or other members of the genus *Homo,* it was controversial. Previously, some archaeologists argued that the spectrum of behaviors in contemporary humans that involve shared meaning emerged as an integrated package in Late Pleistocene Africa, likely related to the dispersal of ‘modern’ *Homo sapiens* throughout the world (e.g. Klein, 1995). This view gave way to a recognition that meaning-laden material was used across a wider span of time, at least across part of the Middle Pleistocene (Mcbrearty and Brooks, 2000), and not only by recent humans. In the last two decades, substantial evidence emerged of the extent of material evidence of meaning-laden behavior attributed to Neanderthals and other members of the genus *Homo* (Kissel and Fuentes, 2018; Kissel and Fuentes, 2021; Colagè and d’Errico, 2025).

**Figure 1.**
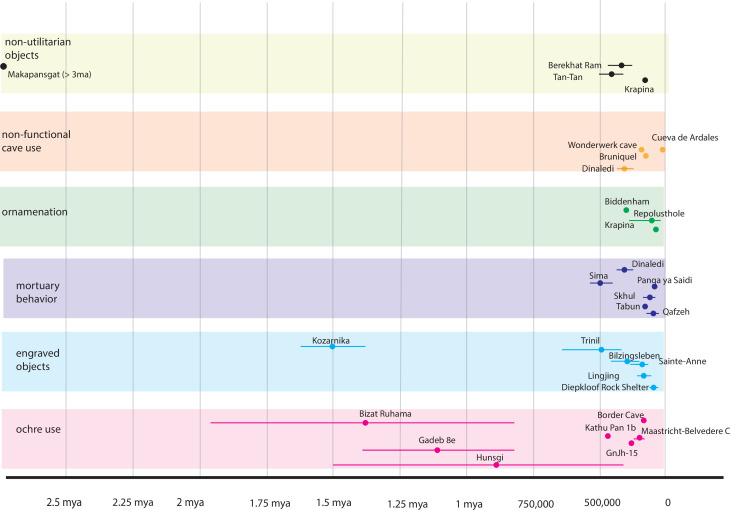
Archaeological evidence of culturally-mediated, meaning-making, behaviors. Dots represent different sites and the error bars are the maximum and minimum dates when available. This table is a sampling of archaeological sites that have been suggested to show signs of what some call ‘symbolic behavior.’ Delimitating what is and what is not symbolic has been the source of contention for many decades now (Habgood and Franklin, 2008; Mcbrearty and Brooks, 2000; Wadley, 2001; Hopkinson, 2013; Deacon, 1997; Anderson, 2012). Traditionally, archaeologists have defined symbols as objects that have meanings embedded in them. Yet a symbol, by its very nature, must be interpreted within a system of meaning and discerning if something is symbolic becomes difficult without knowing the cultural context within which it has been created (Kissel and Fuentes, 2017). We created this table from the published literature to demonstrate that no matter what we choose to call it, culturally-mediated behaviors predate contemporary humans. Such behaviors are found with *Homo erectus* (Joordens et al., 2014), Neandertals (Radovčić et al., 2015) and other archaic populations (d’Errico and Nowell, 2000; Sirakov et al., 2010; Li et al., 2019). Data is taken from Jaubert et al., 2016; Chazan and Horwitz, 2009; Martí et al., 2021; Li et al., 2019; Sirakov et al., 2010; Joordens et al., 2014; Mania and Mania, 1988; Texier et al., 2013; Radovčić et al., 2016; Hovers et al., 1997; Raynal and Seguy, 1986; Bednarik, 2006; Bischoff et al., 2007; Ronen, 1976; Pettitt, 2002; Vandermeersch and Bar-Yosef, 2019; Toro-Moyano et al., 2013; d’Errico and Nowell, 2000; Bednarik, 1998; Bednarik, 2003; Radovčić et al., 2015; Clark and Kurashina, 1979; Ronen et al., 1998; Watts et al., 2016; Roebroeks et al., 2012; Bednarik, 2005; d’Errico et al., 2009; Wadley et al., 2020; Deino and McBrearty, 2002; Berger et al., 2023a; and Berger et al., 2023b. Figure 1—source data 1.These are the data used to construct Figure 1.

Currently, a broad set of data demonstrates that some of these complex behaviors that involve shared meaning were manifested by multiple species and populations of the genus *Homo,* including *Homo heidelbergensis, Homo erectus,* and possibly others (Galway‐Witham et al., 2019; Kissel and Fuentes, 2018; Kissel and Fuentes, 2021; Scerri and Will, 2023; Colagè and d’Errico, 2025; Figure 1). Engravings of shell, bone, or rock surfaces have been identified in Middle Pleistocene or earlier contexts far from the range of African *Homo* populations. Some are also likely associated with *Homo erectus* (Joordens et al., 2014) as well as hominin populations that may have predated early Neanderthals in what is now Europe (Mania and Mania, 1988; Sirakov et al., 2010). Evidence of ochre use occurs in archaeological contexts across Africa and the Levant prior to 350,000 years ago, a time when *H. sapiens* has not yet been identified there (Ronen et al., 1998; Watts et al., 2016; Dapschauskas et al., 2022). The control of fire by hominins is demonstrated in Early and Middle Pleistocene contexts where most researchers accept that *H. erectus* was present (Brain and Sillent, 1988; Alperson-Afil, 2008; Goren-Inbar et al., 2004; Hlubik et al., 2019; MacDonald et al., 2021). Mortuary evidences are claimed in association with hominins that predate or are not *H. sapiens* (Carbonell and Mosquera, 2006). These geographically and temporally varied instances could be the result of taphonomic or dating issues (Püschel et al., 2021), but it is likely, given the increasing diverse temporal and geographic discovery of these behaviors and material that such complex behaviors associated with shared meaning were manifested by multiple populations/species of the genus *Homo* in addition to *Homo sapiens*. But how important was brain size to the evolution of these behaviors?

**Figure 2.**
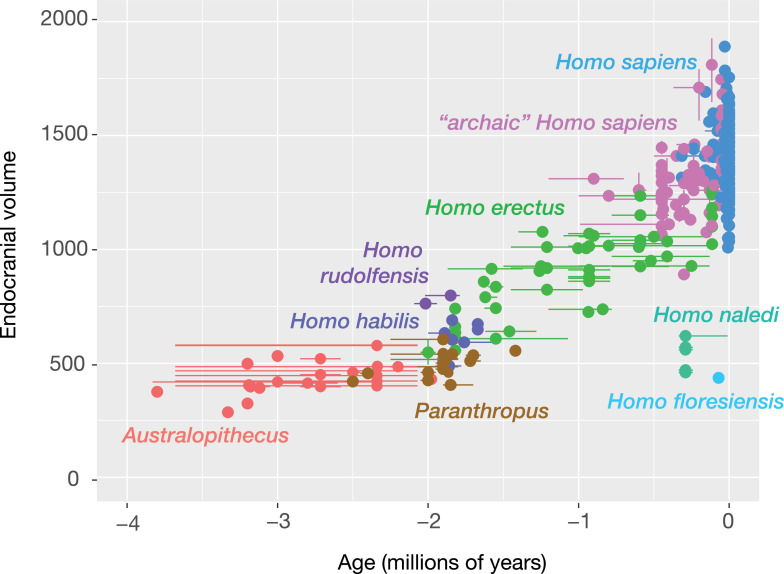
Endocranial volume estimates for hominin cranium. Error bars represent the maximum and minimum ages for specimens when available. See supplemental material for references. Hawks, 2023. Endocranial volumes for fossil hominins (dataset): https://doi.org/10.6084/m9.figshare.22743980.

There is substantive evidence that approximately 250–350,000 years ago, *Homo naledi*, a small-brained hominin, transported deceased conspecifics into difficult to access locations in the Rising Star Cave system in what would, in humans, be described as a mortuary behavior (Berger et al., 2025a). The use of deep areas of the Rising Star cave system for these behaviors implies considerable social collaboration, coordination, and planning. In the context of the subterranean Dinaledi Subsystem, these activities likely also required a light source, again implicating a depth of planning and coordination. What stands out as a possible contradiction is that *Homo naledi* fossil crania are small. With endocranial volumes ranging between 450 ml and 610 ml, this species overlaps in brain size with australopithecines, having smaller brains on average than *Homo erectus* and much smaller than modern humans or Neanderthals (See Figure 2 and section Reconsidering brain size).

The information from *H. naledi* cannot be considered in isolation; it joins the broad array of data for meaning-making in Pleistocene hominins (Kissel and Fuentes, 2018; Kissel and Fuentes, 2021; Malafouris, 2013, Colagè and d’Errico, 2025). These behaviors reflect social groups that maintained solidarity, social coordination, and cooperation in a mode not evident in living great apes but characteristic of contemporary humans. Here, we offer an analysis of the reported complex behavior in the small-brained *Homo naledi* and suggest a suite of implications this has for our understanding of the relationships between brain size, cognition, complex behavior, and the evolution of the genus *Homo* across the Pleistocene. These implications also query the driving forces behind encephalization and its relationship to the emergence of complex behaviors in hominins and other animals (see Tattersall, 2023).

### Mortuary behavior as meaning-making

*Mortuary behavior* has been defined as actions by individuals relating to the death of other individuals. Many kinds of non-human animals have been observed to engage in mortuary behavior upon the death of another individual in the same social group (Piel and Stewart, 2015; Gonçalves and Carvalho, 2019; Pettitt and Anderson, 2020). Mortuary behavior may include the manipulation, inspection, or movement of dead bodies or body parts, or modifying behavior in proximity to dead or dying individuals. Species which manifest strong emotional bonds between individuals, including many primates, elephants, and cetaceans, may continue to interact with a corpse for a period of time after the individual’s death. Many such examples involve mothers who continue to carry a dead infant, often for up to a week after death (Goodall, 1977; Boesch and Boesch-Acherman, 2000; Biro et al., 2010; Fashing et al., 2011), and in some cases other individuals such as unrelated males have been observed to carry or interact with dead infants (Merz, 1978). Species with strong social bonds often exhibit emotional responses to dead or dying individuals, ranging from surprise and fear to prolonged grief (King, 2013). Cannibalism is also a form of mortuary behavior observed in some non-human primates.

Scientific examination of mortuary behavior across living species has been limited. Piel and Stewart, 2015 noted the publication bias related to mortuary behavior in non-human species. Field researchers may hesitate to publish observations that appear to be isolated cases or anecdotes, and as Piel and Stewart point out, almost no studies are negative reports that claim that *no* mortuary behavior occurs in a species. In recent years, a growth in interest in mortuary behavior in nonhuman primates and other social mammals has been fueled by attempts to understand ancestral hominin mortuary practices, including those evidenced in Neanderthals (Pettitt, 2018; Pettitt and Anderson, 2020; Pomeroy et al., 2020). Discussion of the evolutionary background of mortuary behavior in non-human species has focused upon possible adaptive or maladaptive consequences of the behavior (Piel and Stewart, 2015). Less attention has been given to the proximate neural, psychological, or social mechanisms that give rise to mortuary behavior in various species.

There are clear differences between the mortuary behavior repertoire of humans and the mortuary behavior observed in many other living primates. In humans, some mortuary behavior is *ritualized*, meaning that the behavior is systematized and repeated. *Funerary behavior* is a category of mortuary behavior which is defined as specific activities relating to the disposal of the dead and to their subsequent commemoration (Pettitt, 2011; Pettitt, 2018; Pettitt and Anderson, 2020). While funerary behavior is not characteristic of other primates or social mammals, human mortuary behaviors do include many patterns that are also seen in some non-human species, such as the temporary curation of corpses or body parts, disposal of corpses without commemoration, dietary or starvation cannibalism, and manipulation of corpses out of curiosity or fear (Engelke, 2019; Silverman et al., 2021).

To the extent that human behaviors surrounding death are different from non-human primates, those differences reflect primarily two mechanisms. One dynamic underlying human mortuary behavior is the shared, learned cultural traditions concerning death. These traditions vary extensively across human cultures and may include an understanding of the permanence of death, religious beliefs and practices concerning death, and scientific and practical knowledge about the causes of death. When anthropologists examine funerary practices, they often describe this kind of cultural knowledge (Engelke, 2019; Silverman et al., 2021). Another dynamic involved is an evolved emotional cognition, including emotional self-awareness and regulation. In human cultures, these two dynamics are interconnected: religious rituals help grieving individuals by providing social support for emotional regulation and for processing and resolving relational trauma. Superficially similar instances of mortuary behavior in different cultures or species may sometimes involve different proximate mechanisms. For example, ritual and funerary behavior both involve the learned repetition of behavioral and emotional states (Pettitt, 2018; Silverman et al., 2021). In effect, a shared capacity for culture integrates with complex emotional cognition, involving a depth of emotional bonds, capacities for emotional commitments (which necessarily come alongside grief at loss, and a need to process relational trauma), and complex emotional regulation. Both have been practiced by people with entirely different or even incommensurable cultural traditions concerning death, which differ widely, yet fulfil similar emotional needs. Hence, the interpretation of physical evidence for mortuary behavior should consider the range of cultural and cognitive mechanisms that may be at play and how they interact, which may give rise to different equally plausible explanations for the pattern of evidence.

### Rising star evidence and context

The findings from the Rising Star system strongly support a scenario where members of the *H. naledi* community carried the bodies of dead conspecifics to more than 30 meters below the surface, over more than 80 meters of underground passages in a difficult and dangerous subterranean environment (Berger et al., 2025a; Elliott et al., 2021; van Rooyen et al., 2025). The available evidence demonstrates some aspects of mortuary behavior manifested by *H. naledi*. Multiple lines of material evidence show that corpses were manipulated, both at the time of death and afterwards. The distribution of skeletal parts across the Dinaledi Subsystem could not have arisen from deposition at a single point of entry to the subsystem with gravity-driven movement of bodies or bones (Berger et al., 2025a; van Rooyen et al., 2025). Both the spatial arrangements of skeletal material and the form and composition of sediments rule out water flow or mud flow as mechanisms for transport (Dirks et al., 2015; Wiersma et al., 2020; Brophy et al., 2021; Berger et al., 2025a). Movement of remains by *Homo naledi* is the current best hypothesis for the emplacement of bodies or remains. The rapid emplacement of some bodies into sediment prior to decomposition and continued support by sediment through the process of decomposition, together with evidence of disruption of surrounding sediment layering, all suggest that some bodies were interred within shallow holes and covered before soft tissue decomposition occurred (Berger et al., 2025a). Some bodies were manipulated after deposition, as evidenced by the selective reworking of the Puzzle Box area of the Dinaledi Chamber, leading to fragmentation of some skeletal elements, disaggregation of body parts, and commingling of elements from different individuals (Berger et al., 2025a). The presence of young children and infants within the sample likely also reflects the manipulation of bodies by other, presumably older, individuals (Berger et al., 2025a, Delezene et al., 2024).

Many aspects of the mortuary behavior represented by this evidence remain unclear. The evidence does not show whether postmortem manipulation in the Puzzle Box area was deliberate and commemorative in intent, or whether this manipulation was an incidental result of the introduction of subsequent bodies into the Puzzle Box area or other activities in the Dinaledi chamber. Archaeological collection and excavation of remains across the Dinaledi subsystem have revealed varied dispositions of different individuals. Some of the skeletal remains show no evidence of intentional disruption after deposition, while others underwent marked and selective reworking after initial deposition, and other skeletal parts were isolated in long fissure passages (summarized in Berger et al., 2025a). It is unclear whether these differences reflect intentional differences in mortuary behavior, whether they reflect changing traditions over time, or whether they resulted from a lack of precise patterning of mortuary activity. The duration of mortuary activity in the cave system is not known, nor is it known whether the remains in the Lesedi Chamber and in the Dinaledi Subsystem represent activities of the same group, culturally related groups, or unrelated groups. The anatomical evidence also suggests that the sample of *H. naledi* individuals may be biased with overrepresentation of one sex (Delezene et al., 2024), and it is not known whether this bias was an intentional result of the mortuary behavior. While a majority of the remains associated with mortuary behavior of *H. naledi* occur within deep cave areas, it is not clear whether this behavior was limited to these spaces or whether the observed evidence may represent only a small part of a more extensive pattern.

The use of deep cave spaces as part of the mortuary behavior of *H. naledi* provides additional evidence about the social and emotional mechanisms of this species. The subterranean environment used by *H. naledi* is physically challenging for today’s researchers. A longstanding question is whether the system was equally challenging for *H. naledi*. The journey to the Dinaledi subsystem from any known or reasonably hypothesized incursion point involved strenuous scaling and navigation of complex three-dimensional topography across distance, multiple chambers, passages, climbs, and descents (Elliott et al., 2021; Robbins et al., 2021; Berger et al., 2025a:SOM; van Rooyen et al., 2025). To accomplish this, *H. naledi* had to coordinate their behavior and collaborate to move the bodies to a specific location inside the Rising Star cave system. Several aspects of the biology of *H. naledi* suggest that this species may have been better at underground movement than today’s humans: Adult *Homo naledi* individuals had smaller body size than even small-bodied caving team members today (Garvin et al., 2017), and a body plan, including hand morphology, that was more suited for climbing and passing through narrow and restricted cave passages (e.g. Kivell et al., 2015; Feuerriegel et al., 2017; Feuerriegel et al., 2019; Williams et al., 2017; Traynor et al., 2022; Syeda et al., 2025). Still, movement into these spaces would have had high energetic costs and carried some risk for the *H. naledi* individuals undertaking the behavior. Doing so while carrying a corpse would have entailed additional energetic costs. Given the structural complexity of the cave system layout (Berger et al., 2025a, Elliott et al., 2021; Robbins et al., 2021: van Rooyen et al., 2025), there must have been some form of explicit communication (tactile, vocal, and likely visual) for coordination of movement and actions, and the potential use of fire as a light source, between the *H. naledi* undertaking the behavior. Such coordination and specific set of actions around the treatment of deceased conspecifics is more methodologically extensive, energetically costly, with higher risk of injury than any reported for other primates and non-human animals to date (King, 2013). This behavior is also more complex and multifactorial than that reported for the one earlier case of hominin mortuary behavior (Sima de Los Huesos, Carbonell and Mosquera, 2006). There are no clear direct fitness benefits nor any indication of particular proximate functional stimuli for this suite of behaviors.

The subterranean environment used by *H. naledi* is not only physically challenging but is also emotionally and physiologically challenging, reflecting a particular engagement with difficult underground spaces not common in the archaeological record of that time. Dark enclosed spaces, where visual perception is curtailed, can create a state of emotional arousal profoundly affecting perceptual, cognitive, physiological, and social systems (Zuccarelli et al., 2019; Kedar et al., 2021), even with some form of illumination. It is very likely that *H. naledi* used forms of tactile communication and a range of proprioceptive tactics to navigate and communicate in the Rising Star cave system (e.g. Dunbar, 2010; Waller and Hodgson, 2013). However, while such modes of social interactions can enable a range of coordination, it is likely that some form of illumination is also necessary to undertake the behavioral patterns reported (e.g. Pettit, 2022).

In humans, and other diurnal primates, sensory deprivation through reduced or a lack of consistent visual clues creates a heightened sensitivity to other senses (and thus augments proprioceptive dynamics) as well as prompting experiences of visual disturbances, hallucinations, and disorientation (Hodgson, 2021). Experiences of these types of extreme and unusual environments, though often inducing fear responses, can also facilitate powerful bonding experiences (Steidle et al., 2013). Furthermore, the interoceptive nature of bodily awareness, such as that experienced during traversing a complex cave system enhances empathy (Ernst et al., 2013) which may be augmented and deployed in tactile and vocal communication and coordination. This range of substantive emotional, psychological, and physiological reactions may explain why experiences in deep dark caves are often associated with a sense of the transcendent in contemporary humans (Montello and Moyes, 2012; Kedar et al., 2021) and given the broad range of sensory commonalities across diurnal anthropoids, and especially apes, such experiences likely had comparable impacts on *H. naledi* and other Pleistocene hominins using subterranean spaces. We argue that careful and coordinated engagement with the dead on several occasions, in these subterranean environments, implies particularly strong social and emotional bonds and some shared understanding of meaning (Pettitt and Anderson, 2020) in the handling of the dead by *H. naledi*.

As with most examples of Pleistocene mortuary behavior, researchers should be cautious when comparing to modern analogs. Mortuary and funerary behaviors in the past need not map directly to the practices of contemporary or Late Pleistocene humans (see Berger et al., 2025a). As recently suggested by Meneganzin and Killin, 2024: 26 in a discussion of Neanderthal aesthetics: ‘…we should not be surprised if the search for Neanderthal aesthetic practices suggestive of an aesthetic sense requires taking a different route, at least sometimes, to the search for (paradigmatic examples of) human aesthetic practices.’ This suggestion is likely applicable to a range of Pleistocene hominin behavior. An important point of comparison is the Neanderthal use of deep caves, in certain cases reflecting a substantial duration of activity and repeated use (Jaubert et al., 2016; Baquedano et al., 2023).

That this high-risk, high-cost, no-overt-direct-fitness-benefit behavior was undertaken repeatedly by multiple members of an *H. naledi* community indicates a valued, likely cultural, tradition with a social and emotional function. The combination of features in the behavior and the context in which it was undertaken (in deep caves with the likely use of fire for illumination), suggests a level of cognitive/semiotic meaning-making capacity in *H. naledi* (e.g. Kissel and Fuentes, 2017; Kissel and Fuentes, 2018; Kissel and Fuentes, 2021) that matches some similar assessments of other populations of the genus *Homo* during earlier, the same, and later time periods (Figure 1). It is our hypothesis that the repetition of mortuary activities within the Rising Star cave system reflects a form of shared memorialization. This hypothesis emerges from the fact that shared attention and joint action were necessary to generate the evidence within the system, and these actions were repeated over some period of time. The hypothesis of shared memorialization does not depend upon individual interments being repeated with identical steps. What supports the hypothesis is the repeated pattern of cave use and the context and distribution of the remains. The collective practice by *H. naledi,* coupled with social and emotional investment, helped transform the ‘space’ of the Dinaledi Chamber and Hill Antechamber to ‘place’ (Low, 2003) through the pattern of mortuary and possible funerary behavior (e.g. Silverman, 2008).

Some form of mortuary behavior by *H. naledi* is a supported hypothesis (Berger et al., 2025a). However, there is substantive criticism of the assertions of cultural burials in the Dinaledi subsystem (see Berger et al., 2025a, Martinón-Torres et al., 2023; Foecke et al., 2024). Recently, Pettit, 2022 laid out the three key criteria for assessing whether or not the *Homo naledi* remains in the Dinaledi subsystem represent actual funerary behavior: (a) Is there an as-yet unmapped entrance into the Dinaledi Chamber? (b) Is there any evidence of artificial lighting in the cave system, and (c) Is there evidence that it was dead bodies, rather than body parts, that were carried into the chamber? The first query has been repeatedly addressed and no other options for alternative ingress into the Dinaledi Subsystem involving movement across less than 80 meters of structurally complex crevices, chambers, and passages involving a descent to a depth of ~30 meters below the surface have been found or potentially identified (Elliott et al., 2021; Robbins et al., 2021; Berger et al., 2025a; van Rooyen et al., 2025). There is reported evidence of fire use (hearths and charcoal and smoke scarring of surfaces) in the Dinaledi system (see Bower, 2022, but also see Martinón-Torres et al., 2023), but the age of these occurrences has not been reported yet, so its association with *H. naledi* is currently correlational. However, the correlational support is a reasonable hypothesis given there is no evidence across the last decade of investigation of any habitual activity by ancient or recent *Homo sapiens* in the Dinaledi Subsystem orin proximal spaces such as the Dragon’s Back Chamber or Lesedi Chamber. Articulated skeletal elements do make up a substantive percentage of the *H. naledi* remains reported in the Dinaledi system, especially in feature 1 and in the Hill Antechamber feature (Berger et al., 2025a). While the one other major locus of remains in Dinaledi (the Puzzle Box feature and surface collections) has a majority of non-articulated materials, there are some articulated remains present and there is strong evidence that disarticulation resulted from post-depositional reworking, likely by *H. naledi* (Berger et al., 2025a). Therefore, we suggest that the currently available evidence tentatively meets Pettit, 2022 criterion for funerary action. As it stands, the whole of the evidence supports the hypothesis that the *H. naledi* remains in the Dinaledi subsystem are one of the two earliest examples of a mortuary practice in a hominin, and potentially offer the earliest evidence of multiple interments, post-depositional reworking, and thus funerary actions by a hominin.

In addition to the above, the locations, contexts, and the inferred behavior associated with the *H. naledi* remains most likely demonstrate shared meaning-making activity (Kissel and Fuentes, 2017, Kissel and Fuentes, 2018). Certainly, if the remains are post-depositionally reworked, and/or do represent burials, and one accepts the correlational association, and validity, of engravings near the interment sites with *H. naledi* (e.g. Berger, 2025b), they do. But even if one *only* accepts the transport to and placement of bodies in the Dinaledi Chamber and Hill Antechamber locations (and the Lesedi Chamber, Hawks et al., 2017; Berger et al., 2025a) there remains a robust argument for mortuary behavior and the assignation of shared meaning to it. Most documented mortuary and funerary practices have been attributed to *Homo sapiens* and Neanderthals, and aside from the Sima de los Huesos site (Carbonell and Mosquera, 2006) and Dinaledi, most such evidence is later in the Pleistocene (Figure 1 and Table 1). Evidence of funerary behavior is generally assumed to require human-like cognitive capability (Pettitt, 2018). If such behavior is indeed present in a small-brained hominin, it suggests that increases in brain size/EQ are not a necessary precursor for the appearance of complex meaning-making behavior in hominins.

**Table 1.** Table of evidence of potential mortuary behavior in hominins.

Site	Type of site	Multiple bodies	Type	Skeletal age of specimen(s)	Age Estimate (in ka)	Species	Reference
Krems-Wachtberg	open air	yes	burial	infants	31	*Homo sapiens*	Teschler-Nicola et al., 2020
Lake Mungo	open air	yes	burial	adults	40	*Homo sapiens*	Bowler et al., 2003
Taramsa hills	open air	no	burial	child (8–10 yrs old)	45	*Homo sapiens*	Vermeersch et al., 1998
La Ferrasie	cave	yes	burial	children?	45	Neandertal	Gómez-Olivencia et al., 2018
Shanidar	cave	yes	burial	adults, infants, 2–3 yr old	45	Neandertal	Pomeroy et al., 2020
Mezmaiskaya Cave,	cave	no	burial	infant	45	Neandertal	Golovanova et al., 1999
La Chapelle-aux-Saints 1	cave	no	burial	adult	50	Neandertal	Rendu et al., 2014
Dederiyeh Cave,	cave	yes	burial	children	50	Neandertal	Akazawa et al., 1999
Régourdou Cave	cave	no	burial	adult	50	Neandertal	Maureille et al., 2001
Kebara	cave	yes	burial	child and adult	55	Neandertal	Pettitt, 2011
Amud	cave	yes	burial	infant and adults	60	Neandertal	Hovers et al., 2000
Roc de Marsal	cave	no	Burial?	child	70	Neandertal	Maureille and Knüsel, 2022
Panga ya Saidi	cave	no	burial	2.5–3 yrs old	78	*Homo sapiens*	Martinón-Torres et al., 2021
Qafzeh	cave	yes	burial	children and adults	100	*Homo sapiens*	Vandermeersch and Bar-Yosef, 2019
Skhul	cave	yes	burial	adults and children	110	*Homo sapiens*	Ronen, 1976
Tabun	cave	yes	burial	adult (maybe neonate?)	120	Neandertal	Pettitt, 2002
Border Cave	cave	yes	burial	adult and infants	74	*Homo sapiens*	d’Errico and Backwell, 2016
Sima de los huesos	pit	yes	caching/mortuary behavior	adults and children	500	Neandertal	Bischoff et al., 2007
Moula-Guercy	cave	yes	modification/ mortuary behavior?	adults and children			Defleur et al., 1999
Herto	open air	no	modification/ mortuary behavior?	adult	160	*Homo sapiens*	White et al., 2003
El Sidron	cave	yes	modification/ mortuary behavior?	adults and children	480	Neandertal	Rosas et al., 2006
Bodo	open air	no	modification/ mortuary behavior?	adult	600	*Homo sapiens*	White, 1986
Gran Dolina	cave	na	modification/ mortuary behavior?	adult and children	800	*Homo sapiens*	Fernández-Jalvo et al., 1999
Sterkfontein	cave	no	modification/ mortuary behavior?	adult	1635	Australopithecus	Pickering et al., 2000
Krapina	cave	yes	mortuary behavior	many age ranges	130	Neandertal	Russell, 1987
AL-333	open air	yes	mortuary behavior?	adults, juveniles and infants	3200	Australopithecus afarensis	Pettitt, 2011

### A role for emotional cognition?

The achievement of social collaboration and social solidarity in humans relies upon emotional cognition and emotional regulation. A broad array of data supports the hypothesis that emotional regulation and self-awareness were prerequisites of human social behaviors involving solidarity and cooperation, including cultural learning, language, and provision of extended care (Spikins et al., 2019).

Humans share many building blocks of emotional cognition with other mammals, and some complex abilities with other primates. We share the same visceromotor and sensorimotor foundation for emotions with other mammals, for example (Steklis and Lane, 2013). Moreover, a range of common emotional responses in humans has now been documented in other primates, particularly in apes, through measurement of heart rate and skin conductance, as well as more recently pupil mimicry and infrared thermography (Nieuwburg et al., 2021). Interpersonal emotional interactions have a common basis. Emotional contagion is apparent in monkeys and apes, and apes in particular demonstrate a level of empathy through yawning and even sympathy through active consolation (Romero et al., 2010; Preston and de Waal, 2002). Diverse primate species have the cognitive ability to infer emotional meaning from expressions (Nieuwburg et al., 2021). Moreover, there is anecdotal evidence for the foundations of cognitive empathy in targeted helping within apes (Koski and Sterck, 2010).

One important aspect of human emotional cognition is the ability to regulate emotions by bringing feelings into ‘rational’ thought (Green and Spikins, 2020). Humans communicate and engage in shared intentions and meaning-making, to a degree not seen in other animals and demonstrate motivations to share emotions, experiences, and activities with other persons (Steklis and Lane, 2013; Fuentes, 2018). Emotional self-awareness, and with it the capacity to regulate emotions—that is, to calmly tolerate difficult feelings and bring them into rational thought—is key to many human social behaviors. Emotional self-awareness is essential to translating empathy into systematic compassionate helping for others, for example (Spikins, 2022). Furthermore, emotional self-awareness allows the regulation of interpersonal emotional vulnerabilities which foster connection and collaboration, such as fearfulness (Grossmann, 2022). Self-conscious emotions also more broadly regulate social behaviors in general (Beer et al., 2003) and conversely, impaired emotional awareness interferes with normal social function in both clinical and non-clinical populations (Gu et al., 2013). Perhaps most importantly, emotional self-awareness provides the basis for emotional commitments which bring high levels of give and take to social relationships. Capacities to make emotional commitments to group interests are often demonstrated through costly signaling in risky, nonfunctional ways (Hall et al., 2015; Lang and Kundt, 2024).

Another important aspect of human emotional cognition is the presence of emotional cues that are absent or not well developed in other living species. The depth of human emotional commitments comes with costs. First, living humans accentuate emotions associated with social control, including shame and guilt, which are not manifested in similar ways in other great apes (Boehm, 2012; Turner, 2014). Sympathy for others and guilt both emerge early in human ontogeny, with sympathy providing a basis for prosocial orientation in very young children and guilt helping provide motivation for repair of social ruptures (Vaish and Grossmann, 2022). These have sometimes been examined within the framework of *moral emotions*, which would additionally include emotions such as contempt, gratitude, and disgust (Fitouchi et al., 2023). These aspects of emotional cognition in humans provide a foundation for prosociality and social solidarity. Both emotional self-awareness and moral emotions function to regulate social interactions and maintain cohesive and cooperative social relationships. Second, emotional bonds with high degrees of give and take, and emotional commitments, which drive behaviors such as risky hunting or caring behaviors necessarily involve relational trauma at loss, regulated and resolved through social relationships and cultural practices.

The *H. naledi* evidence suggests that a human-style conscious emotional awareness was present in this hominin despite its small brain size. The hominins carrying out these mortuary activities would need to be able to bring their emotions into ‘rational thought’ in order to both be aware of their own grief and communicate and coordinate shared intentions over the bodies of the deceased. Moreover, they would need to be able to regulate their emotions (hold feelings in calm awareness) such that they were able to mutually engage in coordination to carefully negotiate extensive, complex subterranean landscapes, despite the risk and complexity of such behavior, to transport bodies into the Dinaledi Subsystem. This level of emotional regulation, coordination, and awareness is markedly different from the generally personal (such as corpse interaction), sometimes numb (corpse avoidance), and often disordered displays of grief in our nearest relatives (Pettitt and Anderson, 2020). The shared and planned transportation and placement of several bodies in the Rising Star system is also evidence of a shared set of cognitive commitments, beliefs, or assumptions about meaning and action, something similar to what one would term ‘shared grief’ and/or ‘shared belief’ in contemporary humans. The behavioral sequences required for mortuary action also suggest a form of shared memorialization, or at least more behaviorally and communicationally complicated shared attention and action to achieve the deposition of the bodies in the locations in the Rising Star system. Regardless of whether one accepts the interpretation of burials and the presence and association of engravings with *H. naledi*, the underlying cognitive processes associated with just the transport and placement of *H. naledi* into the Dinaledi subsystem indicate a level of conscious emotional awareness that enables and is associated with extensive shared intentionality, forward planning, and repeated cultural behavior involving bodily risk. Equally complex use of caves by Neanderthals Jaubert et al., 2016; Baquedano et al., 2023 demonstrates a similar emotional self-awareness, and production of highly symmetrical stone tools is also potentially indicative of certain aspects of emotional awareness and regulation in earlier members of the genus *Homo* (Green and Spikins, 2020). Furthermore, this evidence suggests a depth of emotional commitments, with a willingness to take risks and costs on another’s behalf. Social understanding of emotions is widely accepted as adaptive in an evolutionary context (Nieuwburg et al., 2021) and emotional awareness is associated with better life outcomes in contemporary human contexts (Smith et al., 2023).

That complex emotional cognition is not unique to *Homo sapiens* should not be surprising, but it is not strictly associated with overall brain size (Hurst, 2017). The fact that a small-brained hominin displays these sorts of behaviors suggests that the neurological capacity enabled by a brain larger than 1000 cc cannot be the only factor, or necessarily the main factor, enabling the kind of emotional cognition that is considered a central factor in human evolutionary success. Particular brain areas are related to emotional regulation, including anterior cingulate cortex (Giuliani et al., 2011) and amygdala (Davidson et al., 2007). Much in the prefrontal cortex has also been implicated in emotional regulation and executive control (Davidson et al., 2007). Recent approaches also focus on large-scale brain networks being implicated in emotional regulation (Morawetz et al., 2020; Pozzi et al., 2021; Rieck et al., 2024). The associations between brain areas or networks and emotional regulation and self-awareness are studied by considering how these cognitive traits correlate with the volumes of various brain areas, and by the activation of brain areas as indicated by oxygen consumption during experimental tasks. Neither kind of study can be compared easily with studies of fossil hominins. The only data on brain anatomy from fossil hominins are the volume of the endocranial cavity and the few sulcal and gyral patterns that imprint on the endocranial surface. These data provide one suggestive indication that emotional regulation may have been important across *Homo*, which is that species of the genus have humanlike frontal cortex configurations, including *Homo floresiensis* and *Homo naledi* (Falk et al., 2005; Holloway et al., 2018; Hurst et al., 2024).

The anatomical data alone do not answer when human-like emotional self-awareness and emotional regulation first evolved. Some have suggested that hominins would have been under selection for these traits early in their evolutionary history because of the need for cooperating, cohesive groups in open habitats with high predation (Turner, 2014). Others have suggested that such behavior is a hallmark of the genus *Homo*, or that abilities such as technical learning or the routine use of fire (Twomey, 2019) could only have been manifested in species with emotional cognition that was similar in ways to recent humans. Green and Spikins, 2020 specifically pointed to several aspects of Early Stone Age artifacts like Acheulean handaxes as possible indicators of self-control, including inhibition and conscious regulation of emotions. The reported *H. naledi* behavioral activities may have depended on emotional commitments to others combined with a set of cultural beliefs/practices, a high level of emotional awareness to manage these, and in turn, collaboration with extensive coordination.

### *Homo naledi* behavior in a broader perspective

The behavior patterns manifested in the Rising Star cave system have a distinctive place within a broader global context of mortuary behavior. The repeated mortuary behavior involving more than 30 individuals from Sima de los Huesos, Spain, is substantially earlier than evidence from the Dinaledi Subsystem (Carbonell and Mosquera, 2006; Arsuaga et al., 2014; Vives, 2015; Sala et al., 2024). Cutmarks on hominin individuals such as the Bodo 1 skull from Bodo, Ethiopia, and the StW 53 skull from Sterkfontein, South Africa, may result from even earlier mortuary activity by hominins (White, 1986; Pickering et al., 2000). In the case of StW 53, the evidence is associated with a skull that many researchers attribute to *Australopithecus,* although the claim of hominin-produced cutmarks has been disputed (Hanon et al., 2018). Curation of hominin skeletal remains was part of mortuary behavior at Herto, Ethiopia, before 160,000 years ago (White et al., 2003; Clark et al., 2003). Archaeological evidence of mortuary behavior becomes increasingly common in later contexts, and within the Late Pleistocene, burials and other kinds of funerary behavior have been attributed to both modern humans and Neanderthals. Within this broader pattern, the Rising Star evidence stands out in two ways: the early possible occurrence of burials (Berger et al., 2025a; Dirks et al., 2015), and the position of *H. naledi* as a phylogenetic outgroup when compared to modern humans and Neanderthals (Dembo et al., 2016; Argue et al., 2017; Caparros and Prat, 2021).

Mortuary behavior is only one category within a broader set of behavior patterns that emerge from shared meaning within social groups (Table 1 and Figure 1). Another category of evidence is the patterned engraving of lines on bones, shells, rocks, or rock walls. Providing geochronological context for such engraved features on rock walls is challenging as it requires that walls themselves be buried in sediment or that engraved features be partially obscured by material susceptible to dating, such as calcite crusts. The purported engraved lines and percussion marks observed within the Dinaledi Subsystem (Berger, 2025b) are not an exception to this challenge; however, the widespread presence of *H. naledi* remains within this space and absence of any evidence of modern human activity other than entry by recent explorers, makes it a reasonable hypothesis that *H. naledi* individuals were authors of these marks. Such a hypothesis is reasonable when considered in a global context. Engraved bones are known from several sites as old or older than the Dinaledi Subsystem, including marked bones, ivory, and stone from Bilzingsleben, Germany (Mania and Mania, 1988), an engraved shell from Trinil, Indonesia (Joordens et al., 2014), and an engraved bone from Kozarnika, Bulgaria, once suggested to be around 1.4 million years old (Guadelli and Guadelli, 2004), although recent geochronological work suggests that the layer is likely early Middle Pleistocene in age (Heydari et al., 2022). A larger array of such evidence is known from later Middle Pleistocene and Late Pleistocene, including examples of engraved hominin bone.

Evidence of the entry into caves by hominins occurs across the span of the fossil record of South Africa, three million years or earlier. What is apparently somewhat unusual in the Rising Star Cave is the repeated hominin use of deep caves, where illumination is necessary, including non-utilitarian use of the space. No evidence of such behavior has yet been found earlier than the Dinaledi Subsystem. Still, the evidence for manipulation of stalagmites and built structures within Bruniquel Cave, France (Jaubert et al., 2016), presumed to have been done by Neanderthals, as well as later Neanderthal marking within deep caves such as Cueva de Ardales, Spain (Martí et al., 2021), or areas far from cave entrances such as at Gorham’s Cave, Gibraltar (Rodríguez-Vidal et al., 2014), brings to mind the activity seen in the Rising Star cave system. Within South Africa, the site of Wonderwerk Cave has evidence that may be contemporary or slightly later than the Dinaledi Subsystem, including ochre and quartz crystals taken approximately 100 m from the cave entrance, although within this linear and large cave, the entrance is always visible from the excavated area (Chazan et al., 2020).

The sustained use of deep cave areas is, in humans, tied to the use of fire for illumination. Hominin control of fire in South Africa long predates the Rising Star evidence, with controlled fire indicated in Member 3 at Swartkrans (only ~800 m from the Rising Star Cave), and in Early Stone Age context at Wonderwerk Cave, by one million years ago (Brain and Sillent, 1988; Brain, 1993; Berna et al., 2012). Both *Paranthropus* and early *Homo* were present in South Africa during that period and occurred in association with combustion evidence (Brain and Sillent, 1988; Brain, 1993). Additionally, cave use by hominins across multiple locations during the period of *H. naledi* and earlier (e.g. Wonderwerk in South Africa and Bruniquel in France) also involved use of fire (Berna et al., 2012; Jaubert et al., 2016). While we cannot yet be certain of the exact modes, intensity, duration, and quality of the fires potentially used by *H. naledi* in the Dinaledi subsystem, it is a strong assumption that they at least provided flickering and moderate intensity light sources.

Use of ochre and other pigments is another category of behavior linked to meaning-making (Dapschauskas et al., 2022; Kissel and Fuentes, 2018). Material evidence of pigments carried or used by hominins has been found at several sites earlier than the Dinaledi Subsystem, including sites within Africa, as well as within southwest Asia and south Asia (Figure 1). Non-utilitarian objects that were transported by hominins into sites include some with physical or iconic resemblance to human figures, including objects from Berekhat Ram, Israel (Goren-Inbar, 1986), and Tan-Tan, Morocco (Bednarik, 2003), both from the later Middle Pleistocene.

This pattern of evidence shows that *H. naledi* and other populations of the genus *Homo* overlap temporally in the expression of meaning-making behavior. The material evidence indicates some degree of shared socioemotional and cognitive processes. Middle Pleistocene hominins varied in brain sizes and cranial and post-cranial morphologies, and many of these varied populations share increased evidence for meaning-making (Figure 1). Such behavior is neither ‘modern’ nor exclusive to larger-brained *Homo sapiens* (and Neanderthals). Whilst this adds further evidence to our understanding of the emergence of hominin cognition, there are also wider evolutionary implications. Much like the evolution of social-emotional abilities in other primates (Nieuwburg et al., 2021), the behavioral evidence for small-brained *H. naledi* may suggest that some degree of analogous and homologous, evolution underlies social emotional complexity in humans.

### Reconsidering brain size

Since the nineteenth century, ideas about the evolution of human behavior have tended to emphasize that larger brains evolved to enable more complex behavior. In the most general sense, this is surely correct. Large brains are expensive to maintain and grow (Isler and Schaik, 2009). Considering these costs, the large brain sizes manifested in some hominin lineages would not have evolved if they were not reliably correlated with survival or reproduction. It is often assumed that a large brain was an essential step towards a uniquely human cognition, social relationships, and culture (Dunbar, 2003; Muthukrishna et al., 2018). Several hominin lineages during the Pleistocene did experience evolution of larger overall and relative brain size, as measured by endocranial volume (Figure 2) and body size. Nothing in the Pleistocene behavioral record refutes the basic idea that such lineage-specific increases in brain size evolved alongside some behavioral adaptations.

But the Pleistocene record today does not support the notion that *every* behavioral adaptation was mediated by overall brain size. Planning and forethought in stone tool production both preceded the first appearance of *Homo* (Harmand et al., 2015). Early populations of *H. erectus* dispersed from Africa into Eurasia before 1.8 million years ago, and the first substantial sample of these hominins has an endocranial volume ranging from 550 ml to 730 ml (Ponce de León et al., 2021). The first bifacial tool traditions were developed before 1.76 million years ago, within a geographic and temporal context where the only extant groups of hominins had small average endocranial volumes (Lepre et al., 2011). The use of fire emerged in excess of a million years ago in Africa (Hlubik et al., 2019) at a time when the average endocranial volume among crania attributed to *H. erectus* was around 750 ml. It is credible that ancestors of *H. naledi* were among these early fire users. The phylogenetic arrangements of species within the genus *Homo* remain uncertain (Hawks et al., 2017; Argue et al., 2017; Dembo et al., 2016; Caparros and Prat, 2021). Still, much evidence suggests that the lineages leading to hominin species with small brain size, including *H. naledi* and *H. floresiensis* emerged from within this early Pleistocene evolutionary context (Dembo et al., 2016; Hawks and Berger, 2020). These species would have been part of the hominin niche that also gave rise to other later Pleistocene members of the genus *Homo* (Mondanaro et al., 2020).

It is also important to be precise about what the small sample of fossils really tells us about differences in brain size between lineages (Figure 2). Known *Homo naledi* individuals for which endocranial volume estimates can be made (n=5) have volumes ranging from 450 ml to 610 ml (Holloway et al., 2018; Hurst et al., 2024), the single known endocranial volume for *H. floresiensis* is 426 ml (Kubo et al., 2013; Falk et al., 2005). With so few fossils, these samples are unlikely to provide an accurate understanding of the average of either species. Moreover, both samples may be biased. The LB1 individual of *H. floresiensis* is inferred to be female based on postcranial morphology (Brown et al., 2004) and male individuals would likely have had larger endocranial volumes. The low degree of variation in size within the *H. naledi* sample suggests that the sample may be biased toward representation of one sex (Delezene et al., 2024), again indicating that the sample average may misrepresent the species mean. Both samples have smaller average endocranial volume than *H. erectus*, yet many of the fossils attributed to this species within Africa have estimated endocranial volumes within or just above the range observed in *H. naledi*, including DNH 134 (Herries et al., 2020), KNM-ER 42700 (Neubauer et al., 2018), DAN5/P1 (Semaw et al., 2020), KNM-OL 45500 (Potts et al., 2004), and OH 12. While global *H. erectus* endocranial volume increased over time (Leigh, 1992), fossils attributed to this species with small endocranial volumes occur across the entire temporal range of the species within Africa.

Different hominin lineages manifested differences in brain organization, and these do not always correspond to changes in brain size (Holloway et al., 2018; Hurst, 2017; Hurst et al., 2024; Ponce de León et al., 2021). Endocast evidence from across the genus *Homo* suggests that humanlike frontal cortex development and morphology occurs across a broader array of species than large brain size (Falk et al., 2005; Holloway et al., 2018; Ponce de León et al., 2021). *Homo floresiensis* and *Homo naledi* both share some humanlike aspects of frontal lobe organization, while they both have brain sizes within the range of *Australopithecus* (Falk et al., 2005; Holloway et al., 2018; Hurst et al., 2024). Early *Homo erectus* is argued to be polymorphic in frontal lobe organization (Ponce de León et al., 2021), and as noted above, many fossil crania attributed to this species have endocranial volumes smaller than 650 ml (Lordkipanidze et al., 2013; Ponce de León et al., 2021; Herries et al., 2020; Semaw et al., 2020).

### Looking forward

The initial rise of complex social behaviors in hominins may have been fueled by evolution of emotional cognition alongside other cognitive processes. Meaning-making by hominins includes a variety of categories of behavior that are joined together by the role of shared intention, repetition, and social collaboration. Mortuary behavior is one of these. Varied expressions of mortuary behavior are present in other social mammals, and hominins are distinct in the extent of repetition, social collaboration, and shared meaning in the process. Rather than relying on increased encephalization and its relation to complex behavior as a *Cognitive Rubicon* in human evolution (see Meneganzin and Currie, 2022), we suggest that a distinctive cultural, empathetic, collaborative niche dependent on increasingly complex and robust relationships between individuals has also been a primary driver in the development of key aspects of human, or human-like, behavior (Galway‐Witham et al., 2019; Mcbrearty and Brooks, 2000; Kissel and Fuentes, 2021; Fuentes, 2017; Spikins, 2022; DeCasien et al., 2022). The increasing data for complex behavior and meaning-making across the Pleistocene should play a major element in structuring how we investigate, explain, and model the origins and patterns of hominin and human evolution (Kissel and Fuentes, 2021; Spikins, 2022; Spikins et al., 2019). The current evidence for *H. naledi* in the Rising Star system pushes back the origins of mortuary, and possibly funerary behaviors, challenges our assumptions about the role and importance of encephalization in human evolution, and suggests that the hominin emotional, socio-cognitive niche is more significant than previously thought.

## Data Availability

Source data has been provided for Figure 1. The following dataset was generated: HawksJ 2023Endocranial volumes for fossil homininsfigshare10.6084/m9.figshare.22743980

## Appendix 1

### Supplementary text for Table 1 on mortuary practices

Table 1 was constructed by searching the literature for examples of funerary behaviors in the Paleolithic. The nature of many of these finds are contentious (Pettitt, 2010). For example, some scholars have rejected Roc-de-Marsal (Sandgathe et al., 2011; Goldberg et al., 2017) as a burial based on reevaluating the context of the site, while others include it in the list of European burials. Similarly, experts are divided as to if La Chapelle-aux-Saints should (Rendu et al., 2014) or should not (Gargett et al., 1989; Dibble et al., 2015) be accepted as an intentional Neandertal burial. We also list sites that are not burials but instead show possible evidence of modifying the body after death. Again, scientists disagree if these are funerary practices or, in the case of cutmarks on hominin bones, cannibalism.

Most archaeologists define burial in a way that lets them detect it archaeologically (excavate a pit, put body in pit, refill pit), but this is only one type of funerary ritual. All cultures must find ways to deal with the beginning of life and with the end of life. How to treat the dead (and how to decide when someone is really dead) is culturally specific. The symbolic practices related to death are also highly varied. Just as with foodways, deathways are mediated by how a culture sees death.

All of this is to say that the symbolic aspects around death are as important as the process of dealing with the body. We might also ask how the death-related symbolic behaviors help people mourn.
