# Supplementary material for: Meaning-making behavior in a small-brained hominin, Homo naledi, from the late Pleistocene: contexts and evolutionary implications
Source: eLife. 2025 Sep 4;12:RP89125. doi: 10.7554/eLife.89125 (PMC12410969; doi:10.7554/eLife.89125)
Supplement: Peer review history

# Editor report: eLife Assessment

**DOI:** 10.7554/eLife.89125.3.sa0

**Authors:** George H Perry

**Affiliations:** Pennsylvania State University United States

**Keywords:** Solid, Valuable

This paper discusses the cognitive implications of potential intentional burial, wall engraving creation, and fire as light source use behaviors by relatively small-brained *Homo naledi* hominins. The discussion presented in the paper is **valuable** theoretically in its healthy questioning of prior assumptions concerning the socio-biological constraints of hominin meaning-making behavior. The discussion also contributes practically given that these behaviors have been ascribed to *Homo naledi* in two associated papers. Still, the strength of evidence in this contribution relies on the validity of the conclusions from the two associated papers, which remain actively questioned. The ultimate assessment of this work will vary among individual readers depending on how they view this debate, but if the conclusions from the associated papers hold up, the conclusions in the current paper can be considered **solid**.

# Referee report: Reviewer #3 (Public review)

**DOI:** 10.7554/eLife.89125.3.sa1

This paper, with a slightly modified title from the initial version, presents the cognitive implications of claims made in two accompanying papers (Berger et al. 2023, 2024) about the creation of rock engravings, the intentional disposal of the dead, and fire use by *Homo naledi*. The importance of the paper, therefore, still relies on the validity of the claims for the presence of socio-culturally complex and cognitively demanding behaviors that are presented in the associated papers. Given the archaeological, hominin, and taphonomic analyses in the associated papers are not adequate to enable the exceptional claims for naledi-associated complex behaviors, the inferences made in this paper are currently incomplete.

The claimed behaviors are widely recognized as complex and even quintessential to *Homo sapiens*. The implications of their unequivocal association with such a small-brained Middle Pleistocene hominin are thus far reaching. Accordingly, the main thrust of the paper is to highlight that greater cognition and complex socio-cultural behaviors were not necessarily associated with a positively encephalized brain. This argument begs the obvious question of whether absolute brain size and/or encephalization quotient (i.e., the actual brain volume of a given species relative the expected brain size for a species of the same average body size) can measure cognitive capacity and the complexity of socio-cultural behaviors among late Middle Pleistocene hominins.

Claims for a positive correlation between absolute and/or relative brain size and cognitive ability are not common in discussions surrounding the evolution of Middle- and Late Pleistocene hominin behavior. Currently, the bulk of the evidence for early complex technological and social behaviors derives from multiple sites across South Africa and postdates the emergence of *H. sapiens* by more than 100,000 years. Such lag in the expression of complex technologies and behaviors within our species renders the brain size-implies-cognitive capacity argument moot. Instead, a rich body of research over the past several decades has focused on aspects related to socio-cultural, environmental, and even the wiring of the brain in order to understand factors underlying the expression of the capacity for greater behavioral variability. In this regard, even if the claimed evidence for complex behaviors among the small-brained naledi populations proves valid, the exploration of the specific/potential socio-cultural, neuro-structural, ecological and other factors will be more informative than the emphasis on absolute/relative brain size.

The paper presents as supporting evidence previous claims for the appearance of similar complex behaviors predating the emergence of our species, *H. sapiens*, although it does acknowledge their controversial nature. It then uses the current claims for the association of such behaviors with *H. naledi* as decisive. Given the inadequate analyses in the accompanying papers, and the lack of evidence for stone tools in the naledi sites, the present claims for the expression of culturally and symbolically mediated behaviors by this small-brained hominin must be adequately established. The importance of the paper thus rests on the validity of the claimed evidence-including contextual aspects-for rock engraving, mortuary practices, and the use of fire presented in the associated two papers.

# Author comment: Author response

**DOI:** 10.7554/eLife.89125.3.sa2

**Authors:** Agustín Fuentes, Marc Kissel, Penny Spikins, Keneiloe Molopyane, John Hawks, Lee R Berger

**Affiliations:** Princeton University Princeton United States; Appalachian State University Boone United States; University of York York United Kingdom; University of the Witwatersrand Johannesburg South Africa; University of Wisconsin-Madison Madison United States; The National Geographic Society Washington DC United States

The following is the authors’ response to the current reviews.

We thank the editors at eLife and the one reviewer for engaging our revised manuscript. As we noted in our previous response to reviewers, which we wrote in October 2024 when we submitted our initial revision the majority of critique we received was targeted not so much at the argument of this manuscript but at the debate regarding the evidence in the two other manuscripts that this one accompanied; “ Evidence for deliberate burial of the dead by *Homo naledi*” and “241,000 to 335,000 Years Old Rock Engravings Made by *Homo naledi* in the Rising Star Cave system, South Africa.” Because of that critique we revised this manuscript to emphasize that the key element in constructing our argument is that *H. naledi* engaged in mortuary behavior (the movement of dead *H. naledi* by living *H. naledi* into the Rising Star cave system) and place that in context of (a) the increasingly complex later Pleistocene record of meaning making activity and (b) the assumed correlations between brain size and cognitive capacities in Pliocene and Pleistocene hominins. This framing, as noted in the eLife editorial comment, is the main thrust of our manuscript.There is a growing convergence of evidence that totality of the currently available data and analyses for *H. naledi* in the Rising Star cave system support mortuary behavior: that is, the agential and intentional action by *H. naledi* individuals in the transport of bodies to the Lesedi Chamber and Dinaledi Subsystem--see Berger et al. 2025 plus the 2nd round reviews and the eLife editorial comment associated with it, and also Van Rooyen et al. 2025. We acknowledge the serious debates around the assertion of *funerary* behavior (cultural burial) and seek to illustrate that while we believe the data support the funerary behavior hypothesis, it is not a necessary requirement for our main argument.

A few specific responses to the reviewer in this revised manuscript:

Reviewer states: “Claims for a positive correlation between absolute and/or relative brain size and cognitive ability are not common in discussions surrounding the evolution of Middle- and Late Pleistocene hominin behavior.” We are not making the argument that absolute brain size in the later Pleistocene is a point of focus, rather that there are many arguments and assertions about EQ and cognitive capacity that are central in the proposals for the evolution of hominins in general and genus Homo in particular across the Plio-pleistocene period. We offer a brief review of this in the text and suggest, as noted by this reviewer, that “exploration of the specific/potential socio-cultural, neuro-structural, ecological and other factors will be more informative than the emphasis on absolute/relative brain size”…this (in their words) is exactly our main point. However, we contend that such a framing should not be exclusive to later Pleistocene contexts, but rather that the examination of earlier hominins might also be better served by moving away from the traditional assumptions of cognitive complexity associated with absolute/relative brain size.The reviewer states: “The authors use, in a number of instances throughout the paper, secondary sources of information such as review papers (e.g., McBrearty & Brooks 2000; Scerri & Will 2023; Galway-Witham et al. 2019) instead of the original works that are the basis for making the desired case.” We do indeed use review papers in the main body of the text for clarity, brevity, and to acknowledge robust previous review work in these areas, however in the supplemental text and with the figures and table we offer substantive bibliographies of the original citations and studies. We encourage readers to please spend time with those materials as well.Finally, the reviewer states: “Given the inadequate analyses in the accompanying papers, and the lack of evidence for stone tools in the naledi sites, the present claims for the expression of culturally and symbolically mediated behaviors by this small-brained hominin must be adequately established.” We are quite specific in this manuscript, and in other publications, that we are not arguing for “symbolically mediated” behavior, but do stand by our non-controversial suggestions of meaning-making, and cultural behavior, as relevant in Pleistocene hominins (e.g. Kissel and Fuentes 2017, 2018). We do not argue that stone tools are necessary as mandatory indicators of such possibilities and lay out the *H. naledi* information in the context of the broader and increasing datasets and analyses for meaning-making behavior in Pleistocene hominins (see Figure 1 and table 1, and in the text).

Our point with this manuscript which we reiterate here is that “The increasing data for complex behavior and meaning-making across the Pleistocene should play a major element in structuring how we investigate, explain, and model the origins and patterns of hominin and human evolution” and we feel that the current evidence for *H. naledi* behavior contributes to the broader suites of data, hypotheses, analyses, and theory building in this endeavor.

The following is the authors’ response to the original reviews (from October 2024).

Before laying out how we addressed the specific comments on this manuscript we want to clarify the goal and intent of this paper to maximize effective critical reading of its contents. We appreciate and look forward to continued critique and enhanced discussion of this topic and argument.

Our starting point for constructing the argument in this manuscript is that *H. naledi* engaged in mortuary behavior. This emerges from the totality of the currently available data and analyses for *Homo naledi* in the Rising Star cave system, which support agential and intentional action by *Homo naledi* individuals in the transport of bodies to the Lesedi Chamber and Dinaledi Subsystem. We do feel that the data support the cultural burial hypothesis as well as the likelihood that at least some of the markings reported as engravings are non-naturally occurring (see Martinón-Torres et al. 2024) and made by *Homo naledi*. But these two elements are not necessary for the validity of the argument we pursue in this manuscript.

Our second key point is that gross brain size does not necessarily correlate with particular patterns of complex behavior in Pleistocene hominins. On this there is wide agreement, yet both scholarly and public arguments for the success of the genus *Homo* and the success of *Homo sapiens* have incorporated an assumption of a Rubicon of cerebral size. From this we propose a third point: that smaller brained Pleistocene hominins, including *Homo naledi*, are part of a Pleistocene hominin niche that includes patterns of complex social and cognitive behavior. Such behavior was historically considered to be exclusive to *Homo sapiens* but is now documented to occur earlier, across a range of hominin taxa in the latter half of the Pleistocene. We offer the case of *H. naledi* behavior in the Rising Star system as an example of this. This case contributes to the development of a broader approach to the cognitive, physiological, and behavioral framings of, and explanations for, Pleistocene hominin behavior.

Responses to specific critiques in the eLife reviews centered on this manuscript:

**Reviewer #1:**

All inferences regarding hominin behaviour and biology of *Homo naledi*, discussed by Fuentes and colleagues, are wholly dependent on the evidence presented in the archaeology preprints being true.

**Reviewer #2:**

Fuentes et al. provide a detailed and thoughtful commentary on the evolutionary and behavioral implications of complex behaviors associated with a small-brained hominin, *Homo naledi*…..While the review by Fuentes et al. highlights important assumptions about the relationship between hominin brain size, cognition, and complex behaviors, the evidence presented by Berger et al. 2023a,b does not support the claim that *Homo naledi* engaged in burial practices or symbolic expression through wall engravings.

**Reviewer #3:**

This paper presents the cognitive implications of claims made in two accompanying papers (Berger et al. 2023a, 2023b) about the creation of rock engravings, the intentional disposal of the dead, and fire use by *Homo naledi*. The importance of the paper, therefore, relies on the validity of the claims for the presence of socio-culturally complex and cognitively demanding behaviors that are presented in the associated papers. Given the archaeological, hominin, and taphonomic analyses in the associated papers are not adequate to enable the exceptional claims for nalediassociated complex behaviors, the inferences made in this paper are currently inadequate and incomplete.

We have clarified in the manuscript text and above why we argue that the inferences we are setting as core to our argument do not require cultural burial or engravings by *H. naledi* be demonstrated. However, we do clarify in the revision that the current evidence for the transport of dead conspecifics into difficult to reach areas deep into the cave system by *naledi* is well supported by the archeological and paleoanthropological data currently available (e.g. Berger et al. 2024, Elliott et al. 2021, Robbins et al. 2021, Hawks et al. 2017) and that this is the basis for our argument.

This is outdated as a review as the 2023 Berger et al, paper is now updated (2025) and one of those reviewers does fully support the burial hypothesis. This reviewer point is incorrect given the current state of the other papers.

**Reviewer #3:**

The claimed behaviors are widely recognized as complex and even quintessential to *Homo sapiens*. The implications of their unequivocal association with such a small-brained Middle Pleistocene hominin are thus far reaching. Accordingly, the main thrust of the paper is to highlight that greater cognition and complex socio-cultural behaviors were not necessarily associated with a positively encephalized brain. This argument begs the obvious question of whether absolute brain size and/or encephalization quotient (i.e., the actual brain volume of a given species relative the expected brain size for a species of the same average body size) can measure cognitive capacity and the complexity of socio-cultural behaviors among late Middle Pleistocene hominins….Claims for a positive correlation between absolute and/or relative brain size and cognitive ability are not common in discussions surrounding the evolution of Middle- and Late Pleistocene hominin behavior.

We assert that claims for a positive correlation between absolute and/or relative brain size and cognitive ability are central—either explicitly or implicitly—in most arguments concerning cognitively complex behavior in the genus *Homo*. This is especially true for ideas about success of Pleistocene *Homo* relative to other hominins. We clarify this in the text offering various citations in support of this position (e.g. Meneganzin and Currie 2022, Galway-Witham, Cole, and Stringer 2019, DeCasien, Barton, and Higham 2022, Dunbar 2003, Kissel and Fuentes 2021, Muthukrishna et al. 2018, Püschelet al. 2021, Tattersall 2023).

**Reviewer #3:**

Currently, the bulk of the evidence for early complex technological and social behaviors derives from multiple sites across South Africa and postdates the emergence of *H. sapiens* by more than 100,000 years. Such lag in the expression of complex technologies and behaviors within our species renders the brain size-implies-cognitive capacity argument moot. Instead, a rich body of research over the past several decades has focused on aspects related to sociocultural, environmental, and even the wiring of the brain in order to understand factors underlying the expression of the capacity for greater behavioral variability. In this regard, even if the claimed evidence for complex behaviors among the small-brained *naledi* populations proves valid, the exploration of the specific/potential socio-cultural, neuro-structural, ecological and other factors will be more informative than the emphasis on absolute/relative brain size.”

While not at all denying the critically important and rich record of cultural complexity in the Late Pleistocene South African archeological record, we disagree that “the bulk of the evidence for early complex technological and social behaviors derives from multiple sites across South Africa and postdates the emergence of *H. sapiens* by more than 100,000 years”. We offer a range of examples and citations in support of our assertion in the text (esp. in pp12-14 and Table 1 and Figure 1)

We lay out the currently available data for such cultural complexity in Figure 1 with extensive documentation and citations for each case in the Supplementary material (both aa a table and a bibliography). We wholly agree with Reviewer 3 that “the exploration of the specific/potential socio-cultural, neuro-structural, ecological and other factors will be more informative than the emphasis on absolute/relative brain size” and are attempting to do just that in the manuscript.

**Reviewer #3:**

The paper presents as supporting evidence previous claims for the appearance of similar complex behaviors predating the emergence of our species, *H. sapiens*, although it does acknowledge their controversial nature. It then uses the current claims for the association of such behaviors with *H. naledi* as decisive. Given the inadequate analyses in the accompanying papers and the lack of evidence for stone tools in the naledi sites, the present claims for the expression of culturally and symbolically mediated behaviors by this small-brained hominin must be adequately established.

We respond to the first part of this critique above (regarding the other papers). But again, we emphasize that although we do feel that the argument for cultural burial is supported (see Berger et al. 2025) what we are arguing for in this paper is that the agential and intentional transportation of dead (mortuary behavior) is the sufficient factor undergirding our proposal. We do not agree that absence of recognizable stone tools at the site negates our proposal and assert that the context provided by Figure 1, and the data in the table for figure 1 in the SOM, in concert with the supported mortuary behavior (transport and emplacement of the dead) offer sufficient support for the argument we make in the text regarding brain size and the role of emotional cognition and complex behavior in the Pleistocene hominin niche and *H. naledi*’s participation in it.
